# Atemwegsmanagement für eine chirurgische Tracheotomie – ein herkömmliches Problem mit ungewöhnlicher Präsentation

**DOI:** 10.1007/s00101-022-01121-y

**Published:** 2022-05-02

**Authors:** Maria-Iulia Crisan, Diego De Lorenzi, Armando Heinle, Thomas Heidegger

**Affiliations:** 1 Departement Anästhesie, Spital Grabs, Spitalregion Rheintal Werdenberg Sarganserland, Spitalstr. 44, 9472 Grabs, Schweiz; 2 Departement Allgemein- und Viszeralchirurgie, Spital Grabs, Spitalregion Rheintal Werdenberg Sarganserland, Spitalstr. 44, 9472 Grabs, Schweiz; 3 Departement für Radiologie, Spital Grabs, Spitalregion Rheintal Werdenberg Sarganserland, Spitalstr. 44, 9472 Grabs, Schweiz; 4 Klinik für Anästhesie und Schmerztherapie, Inselspital, Universitätsspital Bern, Bern, Schweiz

## Anamnese

Ein 71-jähriger, untergewichtiger Patient mit einem BMI von 18,1 kg/m^2^ wurde uns zur Einlage einer PEG-Sonde und Biopsie eines Tumors im Halsbereich in Allgemeinanästhesie zur präoperativen Evaluation zugewiesen. Laut Angaben hatte der Patient in den vergangenen 3 Monaten über 10 kg abgenommen. An narkoserelevanten Begleiterkrankungen waren eine Kardiomyopathie unklarer Genese, ein chronisches Vorhofflimmern und ein ischämischer Schlaganfall im Bereich der linken A. cerebri media bekannt. Aus diesem Grund war der Patient mit einem NOAK antikoaguliert. Anamnestisch war weiter zu erfahren, dass etwa einen Monat früher eine Magnetresonanztomographie abgebrochen werden musste, da es in Rückenlage zu einer ausgeprägten Speichelretention gekommen war und die Bedenken für eine Aspiration zu groß waren. Zu diesem Zeitpunkt war eine Biopsie aufgrund seiner Antikoagulation nicht möglich.

## Klinischer Befund

### Klinische Untersuchung

**Figure 1:**
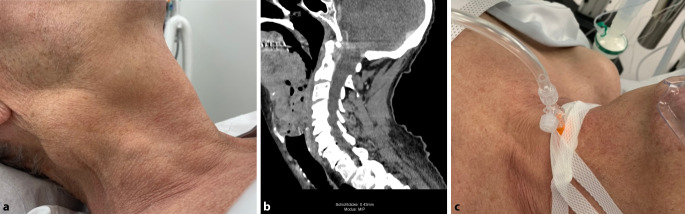

Im Rahmen der präoperativen Visite präsentierte sich der Patient halbsitzend, in einem insgesamt reduzierten Allgemeinzustand, mit einer kloßigen Sprache („Hot-potato“-Stimme); er war tachypnoisch, hatte eine reduzierte Mundöffnung, war unfähig, das Sekret zu schlucken, und hatte sichtlich Angst. Seine Mundöffnung war deutlich eingeschränkt. Ein prominenter, etwa Tischtennisball großer Tumor war auf der rechten Halsseite sichtbar (Abb. 1a). Er wurde als American Society of Anesthesiologists Klasse 3 eingestuft.

### Computertomographie

Die CT zeigte eine große, zentral nekrotische Tumormasse rechts im Niveau des Mundbodens mit Infiltration der Mundbodenmuskulatur, des Zungengrundes sowie des rechtsseitigen Bogens des Os hyoideum, mit einem Durchmesser von ca. 7,0 × 5,5 × 3,5 cm, welche distal zusätzlich die gesamte Epiglottis infiltriert. Die distale Tumorausdehnung reichte bis zur aryepiglottischen Falte und führte zur Verlagerung des Oro- und Hypopharynx sowie des Larynx nach links und resultierte in einer hochgradiger Lumeneinengung (Abb. 1b).

## Behandlung und Verlauf

In Anbetracht der klinischen Situation des Patienten und der raschen Progredienz des Tumors mit drohender Erstickungsgefahr wurde zusammen mit dem Patienten entschieden, eine Tracheotomie durchzuführen.

Eine chirurgische Tracheotomie in Lokalanästhesie wurde vom Patienten aus Angst vor dem Eingriff („Operation am wachen Patienten“) abgelehnt.

Zur Sicherung des Atemweges haben wir uns – im Einverständnis mit dem Patienten – für ein 2‑stufiges Vorgehen entschieden: Erstens, in Lokalanästhesie, Einlage einer infraglottischen Kanüle (Cricath; Fa. Ventinova Medical B.V., Eindhoven, Niederlande) mit einem Innendurchmesser von 2,0 mm zur Sicherung der Oxygenation und, im Falle einer totalen Obstruktion, auch der Möglichkeit einer Ventilation (Abb. 1c). Zweitens, bronchoskopische Intubation (flexibles Intubationsvideoendoskop; Fa Storz, Tuttlingen, Deutschland; Außendurchmesser von 4,0 mm) mit einem Trachealtubus (Teleflex; Fa. Medical Europe Ltd, Athlone, Irland) mit einem Innendurchmesser von 5,5 mm über einen nasalen Zugang. Die hier angewendete Technik der fiberoptischen Intubation ist ein 2‑stufiges Verfahren und wird seit über 30 Jahren beim erwartet schwierigen Atemwegsmanagement erfolgreich durchgeführt [6, 7, 9]: Die nasale Einführung des Bronchoskops und Platzierung in der Trachea erfolgen in rein topischer Anästhesie (Kokain, 10 %, Nasentropfen; Lidocain, 1 %, Spray) ohne Sedation; die normalerweise dazu gehörende transkrikoidale Injektion zur Verabreichung eines intratrachealen Lokalanästhetikums war in dem Fall nicht mehr nötig, da dies bereits im Rahmen der Platzierung der infraglottischen Kanüle erfolgte. Das Vorschieben des Tubus erfolgt erst nach Einleitung der Anästhesie mit Etomidat. Nach definitiver Sicherung des Atemweges wurde die chirurgische Tracheotomie durchgeführt. Im Anschluss wurde die PEG-Sonde eingelegt und eine Biopsie vom Tumor entnommen.

## Diskussion

Die Sicherung des Atemweges für eine Tracheotomie ist ein alltägliches Vorgehen und kann entweder konventionell in Allgemeinanästhesie (mit Videolaryngoskopie) oder mittels fiberoptischer Intubation beim wachen Patienten bewerkstelligt werden [11].

Aufgrund der anatomischen Gegebenheiten (großer supraglottischer Tumor, eingeschränkte Mundöffnung) musste eindeutig von einem erwartet schwierigen Atemweg ausgegangen werden und eine Sicherung des Atemweges nach Einleitung der Narkose (Plan A) kam daher nicht in Betracht [10]. Prinzipiell kommt in so einer Situation entweder eine fiberoptische Intubation beim wachen Patienten oder eine chirurgische Tracheotomie in Lokalanästhesie infrage. Die klassische Wachintubation in rein topischer Lokalanästhesie ohne Sedation (Plan B) lehnte der Patient ab. Die an vielen Orten gängige fiberoptische Intubation mittels der „Conscious-sedation“-Technik unter Erhaltung der Spontanatmung (Plan C) wurde als zu gefährlich betrachtet, da eine zumindest geringfügige Beeinträchtigung der Spontanatmung, die in diesem Fall aber fatal hätte enden könnte, als sehr wahrscheinlich eingestuft wurde [4, 12]. Die Durchführung der Tracheotomie in reiner Lokalanästhesie (Plan D) als Ultima Ratio wurde vom Patienten ebenfalls abgelehnt.

Um auf die speziellen Bedenken des Patienten einzugehen und gleichzeitig auch die klinische Situation zu berücksichtigen, haben wir uns für ein stufenweises Vorgehen entschieden. Wir platzierten zuerst in Lokalanästhesie eine infraglottische Kanüle als Back-up zur Sicherstellung der Oxygenation und auch der Ventilation im Falle einer kompletten Obstruktion des Atemweges. Mittels eines Flow-regulierten, manuellen Gerätes (Ventrain®; Fa. Ventinova Medical B.V.) kann praktisch über einen „Strohhalm“ nicht nur die Inspiration, sondern auch die Exspiration aktiv unterstützt werden [3]. Bei der klassischen Hochfrequenzjetventilation, die ebenfalls über einen transkrikoidalen Katheter durchgeführt und erfolgreich im Management des erwartet schwierigen Atemweges eingesetzt wird [8], muss strikt auf eine permanente Offenhaltung des Atemweges geachtet werden, da es andernfalls sehr rasch zu einem Barotrauma kommen kann [2]. Aus vorher genannten Gründen – Risiko der totalen Obstruktion des Atemweges – haben wir dies daher nicht in Betracht gezogen. Aus denselben Gründen haben wir auch eine Sauerstoffgabe über den Kanal des Bronchoskops nicht in Erwägung gezogen.

Um eine potenzielle Aspiration zu verhindern und auch aus Platzgründen (für die anschließende Tracheotomie) wurde der Patient dann – wie oben detailliert beschrieben – nasal fiberoptisch intubiert.

Dieser Fall verdeutlicht, dass das übliche Vorgehen in bestimmten Situationen angepasst werden muss und eine stufenweise Sicherung der Oxygenation und Ventilation angestrebt werden sollte. Aber oder gerade in solchen Situationen muss vor Beginn gemeinsam ein „plan for failure of the plan“ festgelegt und zwischen allen Beteiligten kommuniziert werden [1, 5]. In unserem konkreten Fall waren dies einerseits die Einlage einer infraglottischen Kanüle als Back-up und andererseits die permanente Anwesenheit eines in der Technik der chirurgischen Koniotomie geübten Chirurgen.

## Fazit für die Praxis

In ausgewählten Fällen ist ein mehrstufiges Vorgehen zur Sicherung des erwartet schwierigen Atemweges indiziert.

Generell muss immer vor Beginn ein „plan for failure of the plan“ festgelegt und zwischen allen Beteiligten kommuniziert werden.

## Abkürzungen

BMI: Body-Mass-Index
CT: Computertomographie
NOAK: Neue orale Antikoagulanzien
PEG: Perkutane endoskopische Gastrostomie
